# Hemoptoe, thin-walled lung cysts, and spontaneous pneumothorax are features of metastatic cutaneous angiosarcoma: A case report

**DOI:** 10.1007/s10354-022-00934-1

**Published:** 2022-05-11

**Authors:** Iurii Mykoliuk, Martin Zacharias, Oliver Sankin, Jörg Lindenmann, Freyja-Maria Smolle-Juettner

**Affiliations:** 1 Division of Thoracic and Hyperbaric Surgery, Medical University Graz, Auenbruggerplatz 29, 8036 Graz, Austria; 2 Diagnostic and Research Institute of Pathology, Medical University of Graz, Neue Stiftingtalstr. 6, 8010 Graz, Austria

**Keywords:** Pulmonary metastasis, Secondary pneumothorax, Hemoptysis, Pulmonary cyst

## Abstract

We present a case of bilateral cystic lung metastases originating from cutaneous angiosarcoma (cAS) of the scalp in a 73-year-old man. He presented with hemoptysis and recurrent bilateral pneumothorax. The clinical, radiological, and histological features and a potential pathophysiological mechanism of pulmonary changes in cutaneous angiosarcoma are discussed.

## Introduction

Cutaneous angiosarcoma is a rare neoplasm that originates from endothelial cells of blood vessels. It accounts for less than 1% of all soft tissue sarcomas and typically develops on the face or scalp [1]. Elderly men are the most affected group. cAS is often associated with either chronic lymphedema or previous irradiation [1, 2].

The lung is the most common site of metastatic involvement in cAS, which typically presents as cystic lesions [3]. The symptoms include hemoptysis [3, 4], pneumothorax [5, 6], pneumomediastinum [7], and hemothorax [6], the latter three being caused by rupture of a cystic metastasis.

Because of the low incidence and the peculiar radiological and clinical presentation, pulmonary metastasis of cAS is often misdiagnosed as mere pulmonary cyst. There are only a few case reports or case series describing cAS affecting the lungs.

We report on a patient with a complex course of pulmonary metastasis of a cutaneous angiosarcoma of the scalp who developed rapidly growing cystic lesions, spontaneous bilateral pneumothorax, intrapulmonary bleeding, and hemoptysis.

## Case report

Two weeks after a first episode of right-sided pneumothorax, which had been treated by chest tube in a peripheral hospital, a 73-year-old male, ex-smoker since 2 years but 60 packyears, presented with recurrent pneumothorax on the right side and slight hemoptoe.

The patient’s history included a parieto-occipital cutaneous angiosarcoma treated by tumor excision and radiation therapy 17 years previously, with local recurrence that had required resection and split-thickness skin grafting 3 years ago. In addition, he had had prostatectomy for carcinoma of the prostate 14 years ago. An outpatient CT scan because of chest pain 8 months previously had shown two thin-walled cysts in the right lower lobe. Despite not taking any anticoagulants, the patient had noticed one episode of slight hemoptysis 3 months ago. The CT scan following that episode had revealed an increase in number of the cysts (four in the right and two in the left lower lobe, with subpleural localization of one cyst on each side), which showed subliminal 18F-fluorodeoxyglucose (FDG) metabolic activity with a standardized uptake value (SUV) of 1.7–2.04. Laboratory and microbiological investigations excluded alpha‑1 antitrypsin deficiency, histiocytosis X, pneumocystis carinii, SARS-CoV‑2, mycobacterial infection. As hemoptysis had subsided spontaneously, the patient had refused any invasive diagnostics.

**Fig. 1:**
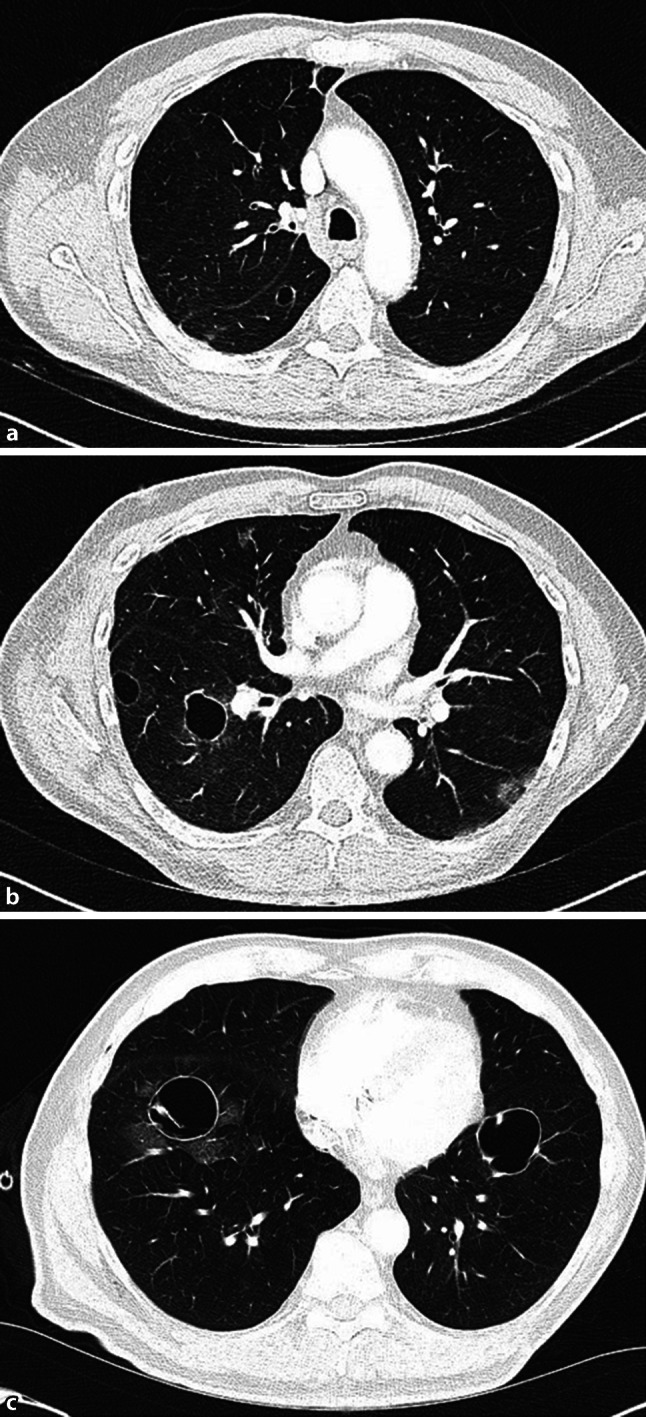
Thin-walled pulmonary cysts in the right and left lower lobes in the **a** upper, **b** middle, and **c** lower lung fields

Thoracic CT scan again showed progression. The bilateral lesions had increased in maximum diameter by 3 to 6 mm each, and measured between 10 and 47 mm (Fig. 1). After chest-tube drainage the lung re-expanded; however, there was a pronounced pulmopleural fistula. During thoracoscopy, a ruptured cyst in the apical segment of the lower lobe was identified and removed by wedge resection.

**Fig. 2:**
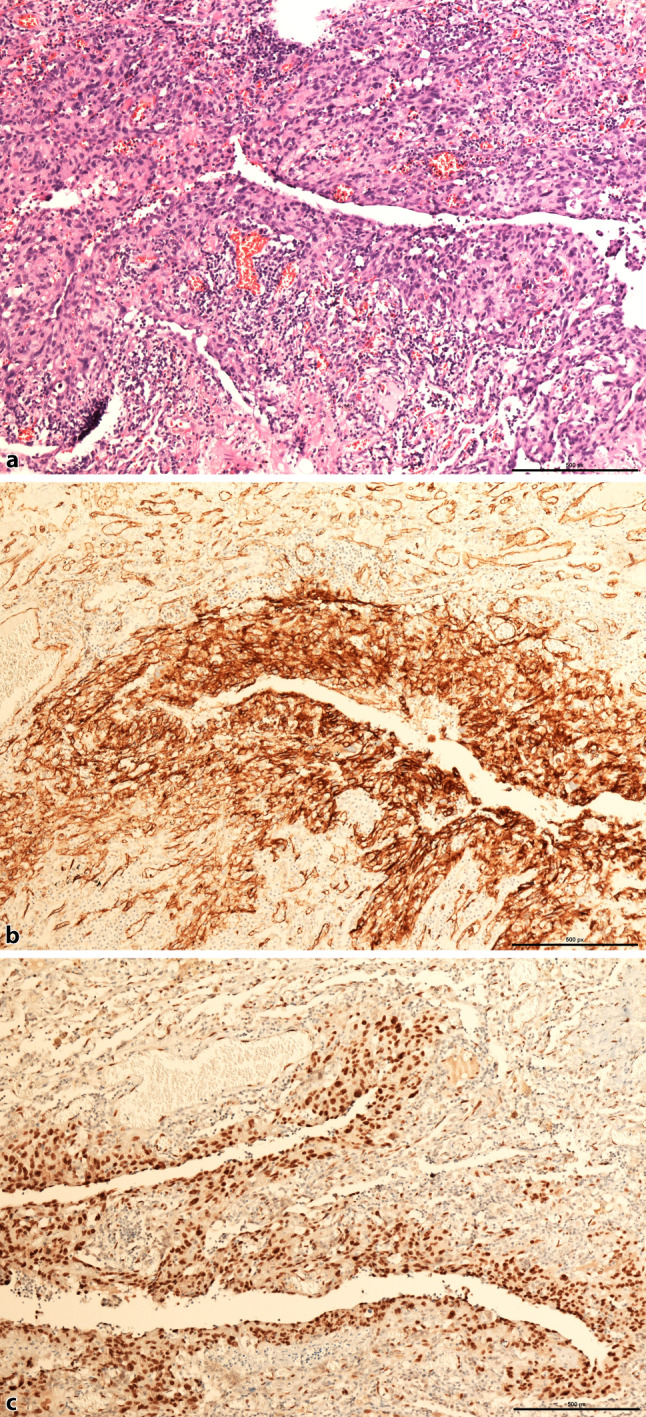
**a** Irregularly shaped anastomosing vascular channels lined by sheets of atypical endothelial cells with an infiltrative growth pattern (H&E stain). Tumor cells demonstrate a membranous positivity for CD31 (**b**) and nuclear positivity for ERG (**c**)

Histopathological workup showed a metastatic spindle cell tumor with CD31 and ERG avian V‑ets erythroblastosis virus E26 oncogene homolog expression, confirming the diagnosis of metastatic angiosarcoma (Fig. 2). *MYC* oncogene was positive in few, singular cells only.

After an uneventful course during which he did not experience further hemoptoe, the patient was discharged on day 5. Since the patient would not have functionally tolerated complete resection of all metastatic lesions, oncological treatment with paclitaxel was initiated.

One month later, the patient was re-admitted with left-sided tension pneumothorax, pronounced hemoptysis, and respiratory insufficiency. The lung re-expanded following chest-tube drainage.

**Fig. 3:**
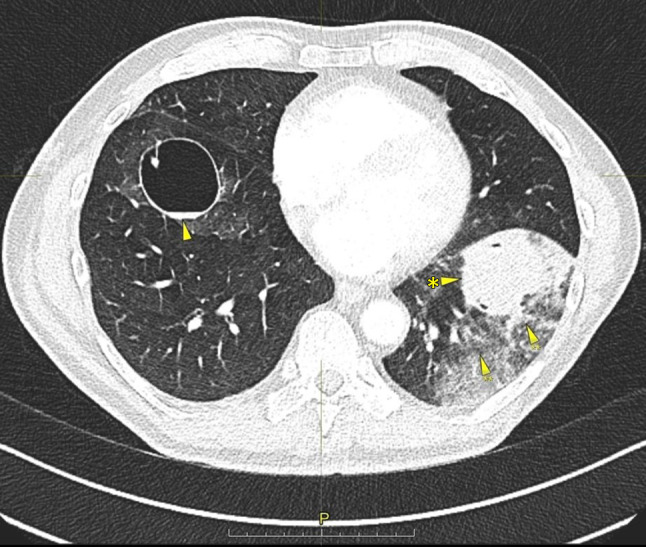
Pulmonary cyst in the right lower lobe (*arrow*), pulmonary liquid-filled cyst (*arrow with asterisk*) in the left lower lobe surrounded by dense intrapulmonary opacity (*arrows with double asterisks*)

**Fig. 4:**
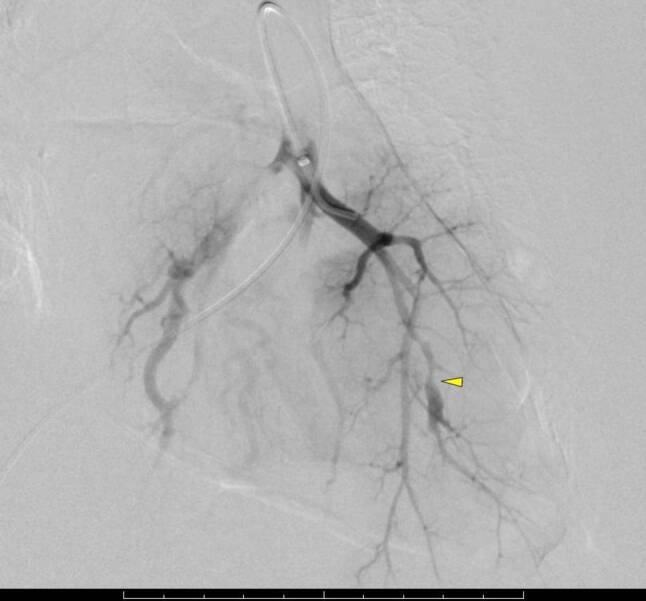
Angiography of the pulmonary artery shows contrast medium extravasation (*arrow*)

**Fig. 5:**
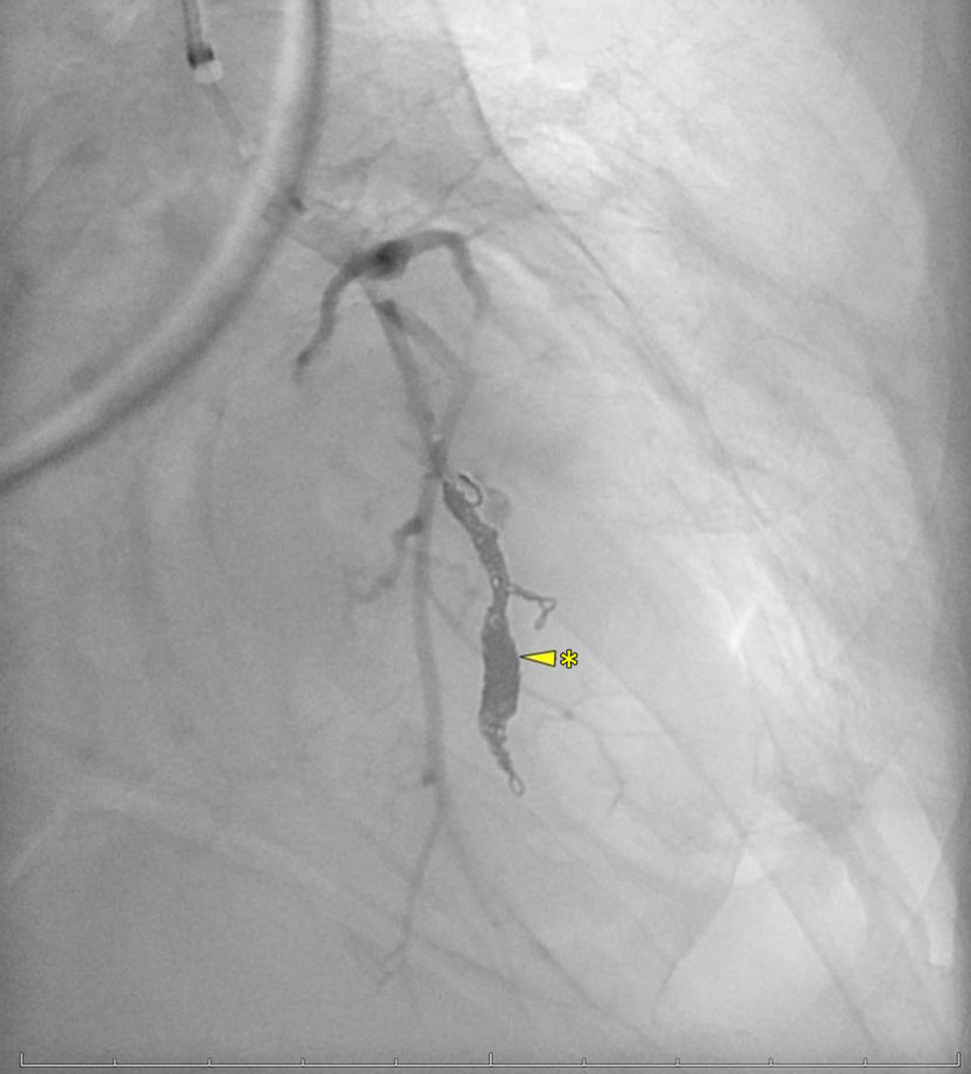
The segmental artery is embolized with coils (*arrow with asterisk*)

Thoracic CT scan revealed another increase in size of the cysts by an average of 5 mm. A large one in the left lower lobe was filled with liquid and surrounded by dense intrapulmonary opacity, suggesting hemorrhage (Fig. 3). By interventional radiology, the segmental pulmonary arteries 8 and 9 on the left side were identified as the source of the bleeding and were coiled, whereupon hemoptysis subsided (Figs. 4 and 5). The chest tube could be removed uneventfully.

Two weeks later, the patient was re-admitted with severe recurrent hemoptysis. The cystic lesion in the left lower lobe had increased by 18 mm in maximum diameter, now comprising the majority of the left lower lobe with the coiling material in unchanged position. Emergency left lower lobectomy was done via thoracotomy. After an uneventful course, the patient was discharged to home care. Up to the time of writing, 1 year after the operation, 10 treatment cycles with paclitaxel (80 mg/m^2^ body surface) have been administered without significant side effects. There has been no further recurrence of pneumothorax or hemoptysis and the patient is up and well.

## Discussion

Cutaneous scalp angiosarcoma (cAS) is a rare, aggressive malignant vascular neoplasm with an incidence of about 0.5 cases per 1,000,000 persons per year [1]. Epidemiological studies indicate that cAS account for less than 1% of all soft tissue sarcomas [1, 8]. They usually occur in males, most of them aged over 60 years [1]. In about 60% of cases, cAS originates in the head and neck region [1].

The lung is the most likely site for metastasis of cutaneous AS [3]. Because of the rare incidence of pulmonary involvement, relevant epidemiological data are not at hand. Only few cases and case series have been reported [3, 5, 9–11]. The clinical presentation, radiological features, and histological pattern of pulmonary metastasis of cAS are characteristic and unique.

In most cases the first symptom of pulmonary spread of cAS is hemoptoe or hemoptysis [1, 4, 12, 13]. Other findings are spontaneous recurrent pneumothorax [3, 11] and rarely hemothorax [14] or pneumomediastinum [7]. The presence of a history of cAS and radiological changes, especially a combination of two or more of these features as found in our patient, should raise the suspicion of pulmonary metastasis. The most peculiar feature in our case of pulmonary metastasis was the long delay of 17 years since initial tumor presentation and a 3-year delay since local recurrence. We were unable to find another report describing such a long interval, since most lung metastases occur synchronously or a few months after treatment of the primary tumor [1, 3, 4].

Radiologically, pulmonary cysts of variable size [3, 6, 10–13] with wall thickness less than 2 mm are typical findings. Additional ground-glass opacities indicate hemorrhage [15, 16]. Due to the thin walls the tumor load is low, and positron-emission tomography (PET) may be misleading since maximum standardized uptake values (SUV) often fail to indicate malignancy. Also, in the present case, PET indicated benign, rather than malignant changes [12].

Histopathology reveals cavities with thin walls, the inner surface of which is lined by tumor cells. They show a diffuse growth pattern, infiltrating the adjacent lung along collagen bundles. Necrosis or fibrosis is usually absent [3, 17]. The tumor cells may be difficult to discern. Immunohistochemical staining including factor VIII, podoplanin, endothelial markers (CD31, CD34, ERG avian V‑ets erythroblastosis virus E26 oncogene homolog), FLI1 (“friend leukemia factor”), and matrix metalloproteinase‑1 (MMP-1) can help to identify cAS in the cyst walls [17, 18]. Our case showed abundant expression of CD31 and ERG avian V‑ets erythroblastosis virus E26 oncogene homolog, whereas *MYC* oncogene was positive in very few, singular cells. Therefore, considering the age of the patient and keeping in mind the fact that the primary had not been radiation induced, the pathologists decided not to proceed to an evaluation of CIC rearrangement. This marker, which indicates poor prognosis, is common in younger individuals and especially in cases associated with radiation therapy [1, 19].

A clear-cut mechanism for the peculiar growth pattern featuring thin-walled cysts has not yet been defined. However, the group of Masuzava et al. suggested a potential explanation. Similar to cells within the cysts in lymphangioleiomyomatosis (LAM), the cAS tumor cells produce matrix metalloproteinase 1 (MMP‑1; collagenase 1). This enzyme is capable of degrading interstitial collagen types 1 to 3 as well as elastin, all of which are components of the alveolar wall. Formation of a central necrosis within a solid metastasis along with a high proteinase activity is another possible mechanism for cyst formation [17, 18]. The specimen of our patient was devoid of any necrosis, however.

Rupture of cystic metastases at the lung surface causing pneumothorax or profuse bleeding may require resection. Yet, as also in our patient, lesions are usually localized deeply within the parenchyma with multiple bilateral spread foci. Therefore, complete resection is usually impossible due to both anatomical and functional reasons. Currently, paclitaxel seems to provide the best systemic treatment option, with long-term tumor control even in case of gross dissemination [20, 21].

## Conclusion

Cutaneous angiosarcoma (cAS) is a rare vascular malignancy. The lung is the typical and most common site of metastasis. Clinical symptoms include hemoptoe, spontaneous recurrent pneumothorax, and rarely hemothorax. Radiologically, multiple thin-walled cysts are found. The present case features the typical symptoms and radiological findings, emphasizing the importance of correct interpretation of pneumothorax and hemoptysis in patients with a history of cAS.
